# Cardiac Function and Structure before and after Mild SARS-CoV-2 Infection in Elite Athletes Using Biventricular and Left Atrial Strain

**DOI:** 10.3390/biomedicines12102310

**Published:** 2024-10-11

**Authors:** Jana Schellenberg, Lynn Matits, Daniel A. Bizjak, Freya S. Jenkins, Johannes Kersten

**Affiliations:** 1 Division of Sports and Rehabilitation Medicine, University Hospital Ulm, 89081 Ulm, Germany; lynn.matits@uni-ulm.de (L.M.); daniel.bizjak@uniklinik-ulm.de (D.A.B.);; 2 Clinical & Biological Psychology, Institute of Psychology and Education, Ulm University, 89081 Ulm, Germany; 3 Medical Faculty, Heinrich-Heine-University Dusseldorf, 40225 Dusseldorf, Germany

**Keywords:** sports cardiology, COVID-19, speckle tracking echocardiography, elite athletes, myocardial inflammation

## Abstract

Background/Objectives: Myocardial involvement has been observed in athletes following SARS-CoV-2 infection. It is unclear if these changes are due to myocardial damage per se or to an interruption in training. The aim of this study was to assess cardiac function and structure in elite athletes before and after infection (INFAt) and compare them to a group of healthy controls (CON). Methods: Transthoracic echocardiography was performed in 32 elite athletes, including 16 INFAt (median 21.0 (19.3–21.5) years, 10 male) before (t_0_) and 52 days after (t_1_) mild SARS-CoV-2 infection and 16 sex-, age- and sports type-matched CON. Left and right ventricular global longitudinal strain (LV/RV GLS), RV free wall longitudinal strain (RV FWS) and left atrial strain (LAS) were assessed by an investigator blinded to patient history. Results: INFAt showed no significant changes in echocardiographic parameters between t_0_ and t_1_, including LV GLS (−21.8% vs. −21.7%, *p* = 0.649) and RV GLS (−29.1% vs. −28.7%, *p* = 0.626). A significant increase was observed in LA reservoir strain (LASr) (35.7% vs. 47.8%, *p* = 0.012). Compared to CON, INFAt at t_1_ had significantly higher RV FWS (−33.0% vs. −28.2%, *p* = 0.011), LASr (47.8% vs. 30.5%, *p* < 0.001) and LA contraction strain (−12.8% vs. −4.9%, *p* = 0.050) values. Conclusions: In elite athletes, mild SARS-CoV-2 infection does not significantly impact LV function when compared to their pre-SARS-CoV-2 status and to healthy controls. However, subtle changes in RV and LA strain may indicate temporary or training-related adaptions. Further research is needed, particularly focusing on athletes with more severe infections or prolonged symptoms.

## 1. Introduction

Worldwide, 772 million people have been infected with severe acute respiratory syndrome-coronavirus-2 (SARS-CoV-2) [1]. The acute phase is primarily characterized by respiratory involvement, but cardiac damage has also been described [2]. Studies in elite athletes have shown that the infection is often mild (46–82%) or asymptomatic (16–58%), with myocarditis occurring only in rare cases (1–3%) [3,4,5]. Most athletes are able to return to competitive and amateur sports after a break from training [6], and longitudinal data also confirm that the majority (96%) of elite athletes are able to continue their professional sports careers after COVID-19 [7].

According to current recommendations, a physical examination and a resting ECG, as well as laboratory tests, should be carried out in the case of moderate or severe disease courses in order to detect myocardial involvement such as myocarditis, pericarditis or cardiac arrhythmia [8] or to shorten the training break after asymptomatic or mild courses [6]. If cardiac symptoms are present, standard transthoracic echocardiography (TTE) is recommended [6], which can be supplemented by the assessment of left ventricular global longitudinal strain (LV GLS), as this is more sensitive for detecting subclinical LV dysfunction than the left ventricular ejection fraction (LV EF) alone [9]. LV and right ventricular (RV) GLS and right ventricular free wall strain (RV FWS) are strong predictors of higher mortality in patients with COVID-19 overall [10,11]. In addition, the assessment of left atrial strain (LAS) can provide further information on LA function, fibrous remodeling [12] and LA contractility [13]. LA strain parameters can predict atrial fibrillation (AF) in COVID-19 patients within the first 48 h of ICU admission [14] and cardiovascular outcomes in non-hospitalized patients [15].

In some studies, no difference in LV GLS was detected between infected athletes and healthy athletes 22 days [16] or 19 days [17] after COVID-19. Conversely, in one of our first studies on this topic, we found significantly reduced LV GLS and diastolic function in a cohort of athletes two months after COVID-19 compared to healthy athletes [18]. In the associated follow-up study, we observed significant improvement in LV GLS approximately five months after COVID-19 [19]. These findings are similar to the follow-up study by Karagodin et al., in which an improvement in LV GLS was observed in patients with impaired baseline function [20]. However, in both our studies, the athlete’s standard echocardiographic parameters and the LV GLS remained within the normal range before and after COVID-19, and the clinical relevance of the changes observed seemed questionable. It is unclear whether these minor changes are due to myocardial damage per se, deconditioning or preexisting subclinical changes. Hence, the primary objective of this study was to assess potential alteration in LV and right ventricular (RV) GLS, global circumferential strain (GCS) and LAS in elite athletes (INFAt) before and after SARS-CoV-2 infection. As a secondary objective, we aimed to determine differences in cardiac function and structure between INFAt after infection and healthy controls (CON).

## 2. Materials and Methods

### 2.1. Study Population

This study was a secondary analysis of the data from the prospective COVID-19 in elite sports study—a multicenter cohort study (COSMO-S), which aimed (1) to analyze the longitudinal rate of seroprevalence of SARS-CoV-2 in German athletes, (2) to assess health-related consequences in athletes infected with SARS-CoV-2, and (3) to reveal the effects of the COVID-19 pandemic in general and those of a cleared SARS-CoV-2 infection on exercise performance [21]. All participants underwent a clinical evaluation that included a medical history and a physical examination, blood sampling, bioimpedance analyses, 12-lead electrocardiogram (ECG) and two-dimensional (2D) TTE and cardiopulmonary exercise testing (CPET).

We analyzed the TTE examinations of 32 elite athletes, including 16 athletes before (t_0_) and after (t_1_) SARS-CoV-2 infection (INFAt) and 16 healthy sex-, age- and sports type-matched athletes (CON) who presented themselves to our institution for their annual check-up. Inclusion criteria were an age between 18 and 35 years, a training volume > 10 h per week in endurance, strength or mixed sports [22], and an elite athlete status, defined as membership in national teams, Olympic squads, or professional sports organizations, alongside a positive SARS-CoV-2 PCR test or positive antibodies with typical COVID-19 symptoms for INFAt. Symptoms during SARS-CoV-2 infection were recorded using questionnaires at presentation. The pre-infection echocardiographic examinations were collected from the respective previous year’s annual check-up (t_0_). All participants provided written informed consent. The exclusion criteria can be found in the study protocol [21] and as described in the previous studies [18,19]. The study was conducted in accordance with the Declaration of Helsinki and approved by the local ethics committee (EK 408/20).

### 2.2. Transthoracic Echocardiography

TTE was performed by an experienced operator using an EPIQ 7 ultrasound system with a phased-array probe X5-1 (Philips GmbH, Hamburg, Germany) according to current international guideline recommendations [23]. The following parameters were assessed: left ventricular end-diastolic and end-systolic volume biplane indexed to body surface area (BSA) (LV EDVi/LV ESVi), left ventricular internal diameter end diastole (LVIDd) and end systole (LVIDs) and both indexed to BSA (LVIDdi/LVIDsi), left ventricular ejection fraction (LV EF by Simpson biplane LV planimetry), left ventricular fractional shortening (LV FS), calculated left ventricular mass (LV mass) using the Devereux formula and indexed to BSA (LV massi), calculated left ventricular stroke volume (LV SV) as a product of LV outflow tract area and outflow tract time-velocity integral, septal thickness (left ventricular interventricular septal end-diastole, LVIVSd) and posterior wall thickness (left ventricular posterior wall end-diastole, LVPWd). M-mode was used in the apical four-chamber view to measure tricuspid annular plane systolic excursion (TAPSE). The size of the left atrium (LA) was quantified in M-mode as LA diameter indexed to BSA (LAi) [23]. Diastolic function was characterized by the E/A ratio (ratio of the mitral valve early—E wave—and late—A wave—inflow velocities), the E/E’lateral ratio (ratio of E wave and E’ of the lateral wall of the mitral annulus) and the E/E’medial ratio (ratio of E wave and E’medial of the septal wall of the mitral annulus) [24]. Heart rate (HR), systolic blood pressure (SBP) and diastolic blood pressure (DBP) were assessed during the physical examination.

### 2.3. Strain Measurements

Strain parameters were determined offline using TomTec postprocessing software (AutoSTRAIN, TomTec Imaging Systems, Unterschleissheim, Germany) by an examiner who was blinded to patient history. LV GLS was obtained in apical four-chamber, two-chamber and long-axis views in the apical, midline, and basal segments [25]. GCS was assessed in short parasternal view in basal and mid segments. RV GLS and RV FWS were obtained in focused RV chamber view. The RV was divided into 3 septal segments (basal, midventricular and apical) and 3 free wall segments (basal, midventricular and apical). RV FWS was determined as the mean value of the free wall segments and RV GLS as the mean value of all 6 RV segments [13,26]. In addition, LAS was determined in the form of LA reservoir strain (LASr), LA conduit strain (LAScd) and LA contraction strain (LASct) in the apical four-chamber view [13,27]. The software automatically tracked the left and right endocardial borders, and a manual adjustment was made to ensure adequate tracking. Normal reference ranges in athletes are not well defined. While according to an expert consensus of the European Association of Cardiovascular Imaging, reference ranges for LV GLS are <−20%, <−23% for RV FWS [28,29] and <−21.5% for RV GLS [30] in the overall population, the reference range for LV GLS in athletes has been identified differently at between −16% and −22% [31].

### 2.4. Statistical Analyses

Statistical analyses were performed using Prism (Version 9.4.1, GraphPad Software Inc., La Jolla, CA, USA, RRID:SCR_002798). The descriptive data are presented as medians and interquartile ranges (IQR) or as absolute values and relative frequencies. Assumptions for linear regression were visually verified using residual plots, QQ plots and histograms. The normal distribution was tested using the D’Agostino–Pearson test. Comparisons were made with a paired *t*-test if the distribution was normal; otherwise, a Wilcoxon signed-rank test was used. A *p*-value of <0.05 was considered significant.

## 3. Results

### 3.1. Cohort Characteristics

A total of 16 INFAt (median age 21.0 (19.3–21.5) years, 10 male) and 16 age-, sex- and sports type-matched CON (median age 20.5 (19.0–22.0) years, 10 male) were included in the statistical analysis. Athletes belonged to the national or state squad, and all practiced endurance, strength or mixed training with a training volume of 10–25 h per week, except for one female short-distance triathlete who showed a training volume of 20–35 h per week. The INFAt and CON groups did not differ in terms of sex, BMI, BSA or resting HR. A significant difference was found between SBP and DBP with higher but still normal values in the CON group (Table 1). The results presented in this study refer to the clinical examination between December 2020 and March 2022, a median of 52 days after SARS-CoV-2 infection (t_1_). Symptoms reported during COVID-19 were fever (31.2%), cough (37.4%), rhinorrhea (56.2%), sore throat (50.0%), resting dyspnea (18.8%) or exertional dyspnea (18.8%) and subjectively perceived reduction in performance (18.8%) compared with maximal performance before COVID-19. Cardiac symptoms were observed in the form of palpitations (6.3%), chest pain (12.5%), increased resting HR (12.5%) and exertional dyspnea (6.3%) (Table 2). All athletes had a mild infection; severe cases, hospitalized athletes or cases of myocarditis were not present in this study.

### 3.2. Echocardiographic Parameters

**Figure 1:**
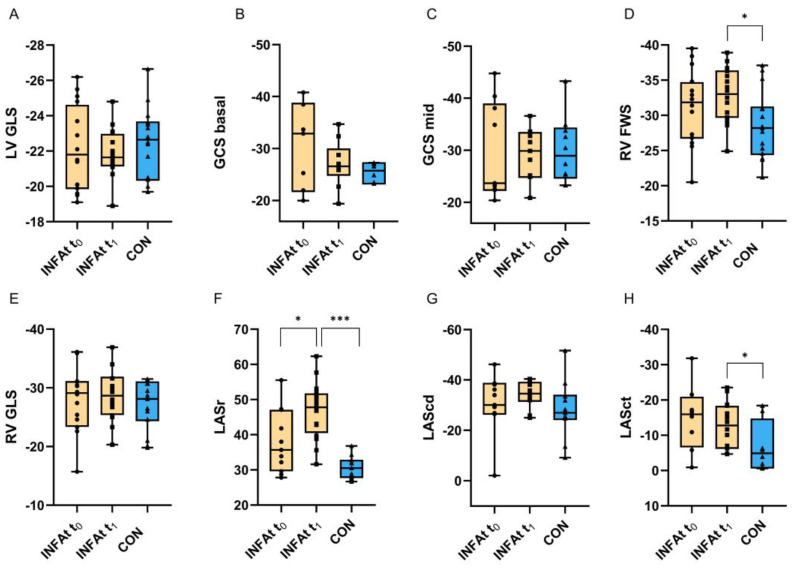
Differences in left and right ventricular strain, circumferential strain and left atrial strain between pre- and post-SARS-CoV-2 infection in athletes (INFAt) and in healthy controls (CON). (**A**): Left ventricular global longitudinal strain (LV GLS) in %; (**B**): Global circumferential strain basal (GCS basal) in %; (**C**): Global circumferential strain midventricular (GCS mid) in %; (**D**): Right ventricular free wall longitudinal strain (RV FWS) in %; (**E**): Right ventricular global longitudinal strain (RV GLS) in %; (**F**): Left atrial reservoir strain (LASr) in %; (**G**): Left atrial conduit strain (LAScd) in %; (**H**): Left atrial contraction strain (LASct) in %. Significant results are presented as follows: * < 0.05; *** < 0.001.

The mean values of LV dimension, LV systolic and diastolic function, and RV function were within the normal echocardiographic reference ranges for adults [23]. None of the standard echocardiographic parameters, nor the LV GLS or the GCS, differed significantly between INFAt and CON (Table 3 and Figure 1). Significant differences were observed between INFAt and CON for RV FWS (INFAt −33.0% vs. CON −28.2%, *p* = 0.011), LASr (INFAt 47.8% vs. CON 30.5%, *p* < 0.001) and LASct (INFAt −12.8% vs. CON −4.9%, *p* = 0.050) with high normal values for INFAt (Table 3 and Figure 1). No significant differences were seen in LV GLS, GCS and RV GLS or RV FWS in INFAt before and after SARS-CoV-2 infection. Conversely, LASr showed higher values at t_1_ in these athletes (t0 35.7% vs. t_1_ 47.8%, *p* = 0.012) (Table 4 and Figure 1).

## 4. Discussion

In this study, elite athletes showed no significant differences in left ventricular cardiac function and structure at a median of 52 days after SARS-CoV-2 infection compared to their pre-SARS-CoV-2 status and to sex-, age- and sports type-matched healthy elite athletes. Minor changes in RV function and LA strain may indicate temporary adaptions. The results indicate that left ventricular cardiac function and structure are not impaired in elite athletes after mild SARS-CoV-2 infection.

### 4.1. LV GLS and GCS

In our study, the mean values of LV dimension, LV systolic and diastolic function, LV GLS and GCS were within the limits of international recommendations [23,30]. High training volumes, particularly in endurance sports, are associated with morphological and functional cardiac adaptations, known as the ‘athlete’s heart’ [32], but these were not observed in our cohort. LV GLS in athletes typically ranges from −16% to −22% [33]. There were no significant differences for LV GLS and GCS between INFAt after SARS-CoV-2 infection and CON. This is in line with studies regarding cardiac function and structure shortly after SARS-CoV-2 infection in elite athletes [16,17].

On the other hand, the number of studies with echocardiographic examinations in elite athletes after SARS-CoV-2 infection is scarce. Most of the results of the studies on this topic relate to hospitalized patients [34,35,36] or to other imaging procedures other than echocardiography, such as cardiac magnetic resonance (CMR) [37,38,39]. In studies with athletes examined with CMR, findings are inconsistent and depend on the size of the study, and are sometimes limited by the fact that there are usually no preliminary examinations or no age-, sex- or sport type-matched comparison groups. It is, therefore, not possible to clarify whether possible changes were triggered by COVID-19 or by training. In some single-center studies, for example, no CMR-defined myocarditis was found in asymptomatic athletes or athletes with mild symptoms [38,39], which corresponds well to our investigated population with a mild course. Echocardiographic studies a few months after infection also show limitations of LV GLS: Gherbesi et al. observed a reduction in LV GLS three months after infection in young adults with an asymptomatic or oligosymptomatic course of disease when compared to a healthy age- and sex-matched control group but, like in our study, the LV GLS remained within the normal range [40]. Interestingly, in a different study, we observed significantly reduced LV GLS and diastolic function in recreational athletes about two months after SARS-CoV-2 infection compared to uninfected healthy athletes [18].

In our previous study, we concluded that although the LV GLS differences were small, they may indicate myocardial involvement after mild SARS-CoV-2 infection in recreational athletes. In this study, with elite athletes, we cannot confirm a potential left myocardial involvement. The precise definition of elite athletes following the condition of having a training volume > 10 h per week, as well as the age-, sex- and sports type-matched classification and, thus, the exclusion of athletes who did not fulfill these criteria from this study, showed that this particular group is less affected by myocardial involvement than recreational athletes. GCS can be used to detect acute myocarditis, assuming subepimyocardial involvement [41]. Transmural myocardial damage can be detected by a reduced GCS as a sign of an advanced stage of the disease [34]. In our population, the GCS was in the normal range, and none of the athletes were diagnosed with myocarditis.

To our knowledge, no other study has yet been able to compare echocardiograms before SARS-CoV-2 infection with those after infection in elite athletes. With the presented echocardiographic examination protocol, we found no significant differences for LV GLS and GCS pre- and post-SARS-CoV-2 infection in elite athletes. The results indicate that elite athletes do not exhibit left ventricular impairment following mild infection. This is indirectly confirmed by a longitudinal study of elite athletes, in which the risk of cardiac effects or clinical events was low, and the majority (96%) of them continued to practice elite sports [7].

### 4.2. RV GLS and RV FWS

RV GLS and RV FWS did not significantly differ in INFAt before and after SARS-CoV-2 infection, and there was also no significant difference between INFAt and CON for RV GLS. This is consistent with the results of previous studies [34,40]. Many studies assessing right ventricular function were performed during acute infection in hospitalized patients. As Li et al. and Xie et al. showed, patients with low RV GLS had a higher HR and a higher incidence of acute cardiac injury and acute respiratory distress syndrome with COVID-19 [10,11]. RV GLS proves to be a good predictor of higher mortality in hospitalized patients with COVID-19 and can, therefore, be used to identify high-risk patients with COVID-19 [10]. In non-hospitalized patients, a decrease in RV GLS and RV FWS was observed after three months in a Turkish [42] and in a Hungarian study [43] 59 ± 33 days after a mild infection. In both studies, the patients were significantly older (approx. 40–44 years) than our elite athletes (median 21 years), and they were not athletes.

In this study, we observed significantly higher RV FWS in INFAt (t_1_) than in CON. As shown in a meta-analysis study, RV FWS is similar between athletes and non-athlete controls, with athletes showing region-specific remodeling, such as lower strain values at the base and higher strain values at the apex [44]. We did not perform a region-specific subdivision in this study. The reason why the RV FWS is significantly lower in the CON group than in INFAt (t_1_) cannot be conclusively explained. RV FWS tends to be higher in physically active people than in sedentary people [45]. Maybe a significantly increased training volume after infection can explain the significantly higher RV FSW in INFAt compared to CON. Alternatively, volume fluctuations at the time of investigation with an additionally small population size can also be considered. No other study so far has examined RV GLS and RV FWS in elite athletes before and after COVID-19. Minor changes in RV function are possibly caused by detraining during the infection. A further aim could be to investigate whether RV FWS can be a marker for subtle changes in cardiac function in elite athletes after a mild infection, even if the LV function remains unchanged.

### 4.3. Left Atrial Strain

In this study, left atrial parameters and diastolic function values were within the limits of the international recommendations [23,27]. LASr and LASct were significantly higher in INFAt than in CON, and LASr was significantly higher in INFAt after infection than before. To our knowledge, no other study has investigated LA strain in elite athletes after COVID-19. Impaired LA strain is described in the setting of an acute SARS-CoV-2 infection in the overall population [46,47]. For instance, ZeinElabdeen et al. showed impaired LASr in 62 patients four to twelve weeks after COVID-19. In their study, LASr and LA stiffness were independent predictors of the development of dyspnea and exercise intolerance after COVID-19 [47]. Even though the participants were of a similar age to our athletes, 31.7% of them still had dyspnea and exercise intolerance, and 33% had sinus tachycardia, which could explain our results. A meta-analysis study showed that LASr and LASct are reduced in endurance athletes [48], which means that pulmonary venous return during LV contraction and isovolumetric relaxation (equivalent to LASr) and passive blood transfer to the LV is reduced (equivalent to LASct) [49]. In contrast, a study by Yaman et al., which investigated the influence of low-intensity sport on LA strain, found higher LASct values in 84 participants with regular sports activity than in 82 sedentary participants [45]. This group presents several differences from our study population, such as older participants, a short training duration (three years) and regular training of around five hours per week, which is significantly less than in our cohort. The current study situation is, therefore, inconsistent. It should be noted that LAS is dependent on age and loading conditions or on LA size, which can make it difficult to compare different study results. Further investigation of LA strain is needed to understand its role during infection, as well as potential temporary or training-induced changes. This warrants additional research, especially focusing on athletes with more severe infections or prolonged symptoms.

### 4.4. Strength and Limitations

First, the strength of this study compared to other COVID-19 observational studies is that the measurements were taken from echocardiograms before and after SARS-CoV-2 infection, allowing to assess changes in cardiac structure and function from the time before or after infection until the study visit. In addition, the time of the study visit after infection may have been long enough for a possible recovery if there were any changes during the acute infection. As we focused on elite athletes between the ages of 18 and 35, we avoided a representation of older adults with potentially more comorbidities and aging conditions, which allowed the assessment of a specific group. Elite athletes after infection were matched with healthy elite athletes according to possible influencing variables such as age, gender and type of sport, so that the groups were well comparable. Cardiac imaging was performed by an experienced operator on one ultrasound machine and analyzed with post-processing software by an experienced examiner blinded to patient history. The study is limited due to the small available number of elite athletes, which is not unusual for single-center studies in sports medicine, and due to the lack of precise data on training volume during or shortly after infection. The restriction to elite athletes with a clearly defined age range also limits the generalizability of the results to the normal population and, perhaps, also to recreational athletes. Of course, the determination of the strain itself has its limitations, such as re-test variability or load dependency and limited assessability due to poor image quality. Another limitation can be that elite athletes with severe SARS-CoV-2 infection or hospitalized athletes were not present in this study, and, therefore, we could not exclude that there would be cardiac structural and functional changes in these groups.

## 5. Conclusions

Elite athletes showed no significant differences in left ventricular cardiac function and structure a median of 52 days after mild SARS-CoV-2 infection compared to their pre-SARS-CoV-2 status and to healthy elite athletes. Therefore, it seems that left ventricular cardiac function and structure are not impaired in elite athletes after mild SARS-CoV-2 infection. Subtle changes in RV and LA strain may indicate temporary or training-related adaptions rather than pathological changes. However, the results of this study regarding LA strain are contradictory and require further investigation. Further research in other directions is also needed, particularly focusing on athletes with more severe infections or prolonged symptoms.

## Figures and Tables

**Table 1:** Cohort characteristics subdivided into athletes infected with SARS-CoV-2 (INFAt) and healthy controls CON). Parameters are given as median and interquartile range (IQR).

	INFAt	CON	Test Statistic	*p*-Value
	Median (IQR)	Median (IQR)		
Number	16	16		
Sex (female/male)	6/10 (37.5%/62.5%)	6/10 (37.5%/62.5%)		
Age (years)	21.0 (19.3–21.5)	20.5 (19.0–22.0)	W = 112	0.555
Weight (kg)	73.5 (59.1–86.5)	73.3 (62.0–76.7)	*t* = 0.26, df = 30	0.799
Height (cm)	183.0 (168.3–189.0)	179.0 (169.8–182.8)	W = 118.5	0.731
BMI (kg/m^2^)	22.4 (20.4–24.9)	22.4 (21.0–23.6)	*t* = 0.21, df = 30	0.834
BSA (g/m^2^)	2.0 (1.7–2.1)	1.93 (1.7–2.0)	*t* = 0.20, df = 30	0.844
Resting HR (bpm)	60.0 (52.8–63.0)	58.5 (53.5–68.8)	*t* = 0.65, df = 30	0.523
Systolic BP (mmHg)	120.0 (110.0–120.0)	125.0 (120.0–137.5)	*t* = 2.45, df = 30	0.020 *
Diastolic BP (mmHg)	70.0 (70.0–80.0)	80.0 (75.0–83.6)	*t* = 2.64, df = 30	0.013 *
Training volume	12–25 h	12–25 h		
Endurance	9	13		
Power	2	2		
Mixed	5	1		

Abbreviations: BMI, body mass index; BSA, body surface area; HR, heart rate; BP, blood pressure. Sports are differentiated according to their predominant component. Based on the 2020 ESC Guidelines on sports cardiology and exercise in patients with cardiovascular disease [22]. Significant results are presented as follows: * < 0.05.

**Table 2:** Symptoms during SARS-CoV-2 infection presented as absolute values and relative frequencies.

Symptoms	N	Present	Not Present
Fever	13	5 (38.5%)	8 (61.5%)
Cough	13	6 (46.2%)	7 (53.8%)
Rhinorrhea	13	9 (69.2%)	4 (30.8%)
Sore throat	13	8 (61.5%)	5 (38.5%)
Resting dyspnea	13	3 (23.1%)	10 (76.9%)
Exertional dyspnea during COVID-19	13	3 (23.1%)	10 (76.9%)
Exertional dyspnea after COVID-19	14	1 (7.1%)	13 (92.9%)
Palpitations	14	1 (7.1%)	13 (92.9%)
Chest pain	14	2 (14.3%)	12 (85.7%)
Increased resting heart rate	14	2 (14.3%)	12 (85.7%)
Subjective perceived performance limitation	14	3 (21.4%)	11 (78.6%)
Dizziness	14	2 (14.3%)	12 (85.7%)

**Table 3:** Echocardiographic parameters subdivided into athletes infected with SARS-CoV-2 (INFAt) and healthy controls (CON). Parameters are given as medians and interquartile ranges (IQR).

	INFAt ^a^	CON ^b^	Test Statistic	*p*-Value
	Median (IQR)	Median (IQR)		
LV EDV (mL)	152.5 (120.5–170.0)	128.5 (103.3–173.5)	*t* = 0.67, df = 28	0.507
LV EDV_i_	77.1 (62.1–83.1)	70.5 (52.1–89.2)	W = 113	0.583
LV ESV (mL)	40.7 (36.9–53.6)	38.8 (28.6–53.4)	W = 86	0.289
LV ESV_i_	22.9 (19.1–25.8)	16.6 (13.2–26.0)	W = 121	0.801
LVIDd (mm)	51.5 (49.3–55.2)	50.6 (46.9–55.8)	*t* = 0.57, df = 30	0.572
LVIDd_i_	27.5 (25.9–29.2)	27.6 (23.9–29.0)	*t* = 0.50, df = 30	0.624
LVIDs (mm)	33.8 (31.8–37.0)	31.7 (27.5–35.9)	*t* = 1.21, df = 30	0.237
LVIDs_i_	18.8 (16.8–19.5)	16.3 (15.3–19.2)	*t* = 1.92, df = 30	0.206
LV EF (%)	62.8 (59.9–65.9)	65.1 (63.2–70.9)	W = 91	0.168
LV FS (%)	34.1 (30.3–36.3)	35.6 (34.5–40.5)	W = 94	0.206
LV Mass (g)	160.0 (143.5–210.5)	166.5 (130.8–227.8)	*t* = 0.20, df = 27	0.839
LV mass_i_ (g/m^2^)	89.9 (55.5–98.6)	86.3 (77.7–113.2)	*t* = 1.68, df = 30	0.104
LVIVSd (mm)	9.0 (8.0–9.8)	9.6 (8.3–11.0)	*t* = 1.73, df = 30	0.945
LVPWd (mm)	8.8 (8.3–10.6)	8.6 (7.7–10.6)	*t* = 0.12, df = 30	0.904
LV SV (mL)	106.0 (81.7–127.5)	93.9 (74.2–121.8)	*t* = 0.34, df = 27	0.739
LV GLS (%)	−21.7 (−22.9–−21.2)	−22.7 (−23.6–−20.4)	*t* = 1.02, df = 30	0.318
GCS basal (%)	−26.6 (−29.7–−25.1)	−25.8 (−27.1–−23.5)	*t* = 0.85, df = 14	0.409
GCS mid (%)	−29.9 (−33.2–−25.1)	−29.0 (−34.1–−24.9)	*t* = 0.27, df = 17	0.788
RV FWS (%)	−33.0 (−36.1–−29.9)	−28.2 (−31.0–−24.6)	*t* = 2.71, df = 29	0.011 *
RV GLS (%)	−28.7 (−31.6–−25.7)	−28.1 (−30.8–−24.6)	*t* = 1.16, df = 29	0.257
TAPSE (mm)	25.6 (21.5–30.5)	25.3 (22.8–27.5)	W = 121	0.802
LA (mm)	32.5 (28.6–35.8)	33.5 (30.0–37.0)	*t* = 1.02, df = 30	0.315
LA_i_	17.3 (15.6–18.9)	18.1 (16.1–20.3)	*t* = 1.01, df = 30	0.321
LASr (%)	47.8 (40.5–51.8)	30.5 (27.7–32.9)	*t* = 6.37, df = 25	<0.001 ***
LAScd (%)	−34.6 (−38.6–−32.0)	−27.0 (−33.5–−24.8)	*t* = 1.87, df = 25	0.074
LASct (%)	−12.8 (−17.9–−6.7)	−4.9 (−14.3–−1.8)	*t* = 2.08, df = 22	0.050 *
E/A	1.7 (1.3–2.2)	1.5 (1.4–1.9)	W = 115	0.635
E/E’l	5.0 (4.7–5.4)	4.8 (4.0–5.2)	W = 118	0.717
E/E’m	6.5 (5.4–7.3)	6.9 (6.5–7.9)	*t* = 1.86, df = 30	0.072
Dec Time (ms)	156.0 (131.8–265.8)	182.0 (143.0–224.0)	*t* = 0.20, df = 21	0.840

Note. ^a^ 16 athletes ^b^ 16 athletes. Abbreviations: W, two-tailed unpaired Wilcoxon Signed Rank Test; T, two-tailed unpaired *t*-test; LV EDV, left ventricular end-diastolic volume; LV EDVi, left ventricular end-diastolic volume/body surface area (BSA); LV ESV, left ventricular end-systolic volume; LV ESVi, left ventricular end-systolic volume/BSA; LVIDd, left ventricular internal diameter end diastole; LVIDd_i_, left ventricular internal diameter end diastole/BSA; LVIDs, left ventricular internal diameter end systole; LVIDs_i_, left ventricular internal diameter end systole/BSA; LV EF, left ventricular ejection fraction by Simpson; LV FS, left ventricular fractional shortening; LV mass, left ventricular mass; LV mass_i_, left ventricular mass/BSA; LVIVSd, left ventricular interventricular septal end diastole; LVPWd, left ventricular posterior wall end diastole; LV SV, left ventricular stroke volume; LV GLS, left ventricular global longitudinal strain; GCS, global circumferential strain; GCS mid, global circumferential strain midventricular; RV FWS, right ventricular free wall longitudinal systolic strain; RV GLS, right ventricular global longitudinal strain; TAPSE, tricuspid annular plane systolic excursion; LA, left atrium; LA_i_, left atrium/BSA; LASr, left atrial reservoir strain; LAScd, left atrial conduit strain; LASct, left atrial contraction strain; E/A, E/A ratio; E/E’l, E/E’l ratio; E/E’m, E/E’m ratio; Dec Time, deceleration time. Significant results are presented as follows: * < 0.05, *** < 0.001.

**Table 4:** Echocardiographic parameters pre- and post-SARS-CoV-2 infection presented as medians and IQR.

	t_0_ ^a^	t_1_ ^b^	Test Statistic	*p*-Value
	Median (IQR)	Median (IQR)		
HR (bpm)	68 (56–70)	60 (52.8–63)	*t* = 1.01, df = 30	0.320
Systolic BP, mmHg	112.5 (110–123.8)	120 (110–120)	*t* = 0.15, df = 30	0.878
Diastolic BP, mmHg	75 (70–80)	70 (70–80)	*t* = 0.80, df = 30	0.430
LV EDV (mL)	131 (119–155)	152.5 (120.5–170)	*t* = 0.82, df = 23	0.421
LV EDV_i_	69.2 (60.3–77.2)	77.1 (62.1–83.1)	W = 95	0.972
LV ESV (mL)	36.9 (30.5–50.1)	40.7 (36.9–53.6)	*t* = 1.00, df = 22	0.328
LV ESV_i_	18.7 (16.3–26.3)	22.9 (19.1–25.8)	W = 80	0.706
LVIDd (mm)	51.6 (50.3–53.8)	51.5 (49.3–55.2)	*t* = 0.04, df = 30	0.965
LVIDd_i_	28.3 (25.4–29.3)	27.5 (25.9–29.2)	*t* = 0.24, df = 30	0.815
LVIDs (mm)	34 (32.6–37)	33.8 (31.8–37)	*t* = 0.03, df = 30	0.973
LVIDs_i_	18.4 (16.5–19.2)	18.8 (16.8–19.5)	*t* = 0.09, df = 30	0.925
LV EF (%)	64.5 (62.1–68.3)	62.8 (59.9–65.9)	W = 111	0.539
LV FS (%)	35.3 (32.9–38.1)	34.1 (30.3–36.3)	*t* = 0.88, df = 28	0.387
LV mass (g)	174 (154–222.8)	160 (143.5–210.5)	*t* = 0.36, df = 25	0.718
LV mass_i_ (g/m^2^)	97.7 (88.4–105.6)	89.9 (55.5–98.6)	W = 75	0.130
LVIVSd (mm)	9.6 (8.5–10.9)	9 (8– 9.8)	*t* = 1.82, df = 30	0.078
LVPWd (mm)	9.6 (8.4–10.9)	8.8 (8.3–10.6)	*t* = 0.89, df = 30	0.382
LV SV (mL)	98.7 (82.9–100)	106 (81.7–127.5)	W = 37	0.523
LV GLS (%)	−21.8 (−24.5–−19.9)	−21.7 (−22.9–−21.2)	*t* = 0.46, df = 30	0.649
GCS basal (%)	−32.9 (−38.5–−22)	−26.6 (−29.7–−25.1)	W = 26	0.403
GCS mid (%)	−23.7 (−38.7–−22.6)	−29.9 (−33.2–−25.1)	*t* = 0.04, df = 17	0.972
RV FWS (%)	−31.9 (−34.5–−26.9)	−33 (−36.1–−29.9)	*t* = 1.00, df = 30	0.326
RV GLS (%)	−29.1 (−30.9–−23.6)	−28.7 (−31.6–−25.7)	*t* = 0.49, df = 30	0.626
TAPSE (mm)	26.7 (22.5–30.5)	25.6 (21.5–30.5)	*t* = 0.06, df = 30	0.950
LA (mm)	34 (31–40.2)	32.5 (28.6–35.8)	*t* = 1.53, df = 30	0.135
LA_i_	19.1 (16.1–20.3)	17.3 (15.6–18.9)	*t* = 1.59, df = 30	0.122
LASr (%)	35.7 (29.6–47.1)	47.8 (40.5–51.8)	*t* = 2.73, df = 25	0.012 *
LAScd (%)	−30.1 (−38.2–−26.9)	−34.6 (−38.6–−32)	W = 67	0.312
LASct (%)	−15.9 (−20.4–7.1)	−12.8 (−17.9–−6.7)	*t* = 0.74, df = 22	0.469
E/A	1.7 (1.5–2)	1.7 (1.3–2.2)	*t* = 0.28, df = 29	0.785
E/E’l	4.3 (4.1–6)	5 (4.7–5.4)	W = 104	>0.999
E/E’m	6.3 (5.6–7.7)	6.5 (5.4–7.3)	*t* = 0.81, df = 28	0.423
DecTime (ms)	162 (132–197)	156 (131.8–265.8)	W = 33	0.469

Note. ^a^ 16 athletes ^b^ 16 athletes. Abbreviations: t_0_, before SARS-CoV-2 infection; t_1_, after SARS-CoV-2 infection; IQR, interquartile range; HR, heart rate; Bpm, beats per minute; BP, blood pressure. The remaining abbreviations are identical to those used in Table 3 and are defined in the abbreviations section there. Significant results are presented as follows: * < 0.05.

## Data Availability

The data underlying this article will be shared on reasonable request to the corresponding author.

## References

[1] WHO (2023). WHO Coronavirus (COVID-19) Dashboard. https://covid19.who.int.

[2] Akhmerov A., Marban E. (2020). COVID-19 and the Heart. Circ. Res..

[3] Moulson N., Petek B.J., Drezner J.A., Harmon K.G., Kliethermes S.A., Patel M.R., Baggish A.L., Outcomes Registry for Cardiac Conditions in Athletes Investigators (2021). SARS-CoV-2 Cardiac Involvement in Young Competitive Athletes. Circulation.

[4] Martinez M.W., Tucker A.M., Bloom O.J., Green G., DiFiori J.P., Solomon G., Phelan D., Kim J.H., Meeuwisse W., Sills A.K. (2021). Prevalence of Inflammatory Heart Disease Among Professional Athletes with Prior COVID-19 Infection Who Received Systematic Return-to-Play Cardiac Screening. JAMA Cardiol..

[5] Clark D.E., Parikh A., Dendy J.M., Diamond A.B., George-Durrett K., Fish F.A., Slaughter J.C., Fitch W., Hughes S.G., Soslow J.H. (2021). COVID-19 Myocardial Pathology Evaluation in Athletes with Cardiac Magnetic Resonance (COMPETE CMR). Circulation.

[6] Steinacker J.M., Schellenberg J., Bloch W., Deibert P., Friedmann-Bette B., Grim C., Halle M., Hirschmüller A., Hollander K., Kerling A. (2022). Recommendations for Return-to-Sport after COVID-19: Expert Consensus. DZSM.

[7] van Hattum J.C., Daems J.J.N., Verwijs S.M., Wismans L.V., van Diepen M.A., Groenink M., Boekholdt S.M., Planken R.N., van Randen A., Hirsch A. (2024). Long-term cardiac follow-up of athletes infected with SARS-CoV-2 after resumption of elite-level sports. Heart.

[8] Writing C., Gluckman T.J., Bhave N.M., Allen L.A., Chung E.H., Spatz E.S., Ammirati E., Baggish A.L., Bozkurt B., Cornwell W.K. (2022). 2022 ACC Expert Consensus Decision Pathway on Cardiovascular Sequelae of COVID-19 in Adults: Myocarditis and Other Myocardial Involvement, Post-Acute Sequelae of SARS-CoV-2 Infection, and Return to Play: A Report of the American College of Cardiology Solution Set Oversight Committee. J. Am. Coll. Cardiol..

[9] Smiseth O.A., Torp H., Opdahl A., Haugaa K.H., Urheim S. (2016). Myocardial strain imaging: How useful is it in clinical decision making?. Eur. Heart J..

[10] Li Y., Li H., Zhu S., Xie Y., Wang B., He L., Zhang D., Zhang Y., Yuan H., Wu C. (2020). Prognostic Value of Right Ventricular Longitudinal Strain in Patients with COVID-19. JACC Cardiovasc. Imaging.

[11] Xie Y., Wang L., Li M., Li H., Zhu S., Wang B., He L., Zhang D., Zhang Y., Yuan H. (2020). Biventricular Longitudinal Strain Predict Mortality in COVID-19 Patients. Front. Cardiovasc. Med..

[12] Cameli M., Mandoli G.E., Loiacono F., Sparla S., Iardino E., Mondillo S. (2016). Left atrial strain: A useful index in atrial fibrillation. Int. J. Cardiol..

[13] Badano L.P., Kolias T.J., Muraru D., Abraham T.P., Aurigemma G., Edvardsen T., D’Hooge J., Donal E., Fraser A.G., Marwick T. (2018). Standardization of left atrial, right ventricular, and right atrial deformation imaging using two-dimensional speckle tracking echocardiography: A consensus document of the EACVI/ASE/Industry Task Force to standardize deformation imaging. Eur. Heart J. Cardiovasc. Imaging.

[14] Beyls C., Hermida A., Bohbot Y., Martin N., Viart C., Boisgard S., Daumin C., Huette P., Dupont H., Abou-Arab O. (2021). Automated left atrial strain analysis for predicting atrial fibrillation in severe COVID-19 pneumonia: A prospective study. Ann. Intensive Care.

[15] Todaro M.C., Choudhuri I., Belohlavek M., Jahangir A., Carerj S., Oreto L., Khandheria B.K. (2012). New echocardiographic techniques for evaluation of left atrial mechanics. Eur. Heart J. Cardiovasc. Imaging.

[16] Lakatos B.K., Tokodi M., Fabian A., Ladanyi Z., Vago H., Szabo L., Sydo N., Csulak E., Kiss O., Babity M. (2021). Frequent Constriction-Like Echocardiographic Findings in Elite Athletes Following Mild COVID-19: A Propensity Score-Matched Analysis. Front. Cardiovasc. Med..

[17] Fikenzer S., Kogel A., Pietsch C., Lavall D., Stobe S., Rudolph U., Laufs U., Hepp P., Hagendorff A. (2021). SARS-CoV2 infection: Functional and morphological cardiopulmonary changes in elite handball players. Sci. Rep..

[18] Schellenberg J., Ahathaller M., Matits L., Kirsten J., Kersten J., Steinacker J.M. (2023). Left Ventricular Global Longitudinal Strain as a Parameter of Mild Myocardial Dysfunction in Athletes after COVID-19. J. Cardiovasc. Dev. Dis..

[19] Schellenberg J., Matits L., Bizjak D.A., Kersten J., Kirsten J., Vollrath S., Steinacker J.M. (2023). Assessment of myocardial function and cardiac performance using left ventricular global longitudinal strain in athletes after COVID-19: A follow-up study. Front. Cardiovasc. Med..

[20] Karagodin I., Singulane C.C., Descamps T., Woodward G.M., Xie M., Tucay E.S., Sarwar R., Vasquez-Ortiz Z.Y., Alizadehasl A., Monaghan M.J. (2022). Ventricular Changes in Patients with Acute COVID-19 Infection: Follow-up of the World Alliance Societies of Echocardiography (WASE-COVID) Study. J. Am. Soc. Echocardiogr..

[21] Niess A.M., Widmann M., Gaidai R., Golz C., Schubert I., Castillo K., Sachs J.P., Bizjak D., Vollrath S., Wimbauer F. (2022). COVID-19 in German Competitive Sports: Protocol for a Prospective Multicenter Cohort Study (CoSmo-S). Int. J. Public Health.

[22] Pelliccia A., Sharma S., Gati S., Back M., Borjesson M., Caselli S., Collet J.P., Corrado D., Drezner J.A., Halle M. (2021). 2020 ESC Guidelines on Sports Cardiology and Exercise in Patients with Cardiovascular Disease. Rev. Esp. Cardiol. (Engl. Ed.).

[23] Lang R.M., Badano L.P., Mor-Avi V., Afilalo J., Armstrong A., Ernande L., Flachskampf F.A., Foster E., Goldstein S.A., Kuznetsova T. (2015). Recommendations for cardiac chamber quantification by echocardiography in adults: An update from the American Society of Echocardiography and the European Association of Cardiovascular Imaging. Eur. Heart J. Cardiovasc. Imaging.

[24] Dalen H., Thorstensen A., Vatten L.J., Aase S.A., Stoylen A. (2010). Reference values and distribution of conventional echocardiographic Doppler measures and longitudinal tissue Doppler velocities in a population free from cardiovascular disease. Circ. Cardiovasc. Imaging.

[25] Voigt J.U., Pedrizzetti G., Lysyansky P., Marwick T.H., Houle H., Baumann R., Pedri S., Ito Y., Abe Y., Metz S. (2015). Definitions for a common standard for 2D speckle tracking echocardiography: Consensus document of the EACVI/ASE/Industry Task Force to standardize deformation imaging. J. Am. Soc. Echocardiogr..

[26] Mor-Avi V., Lang R.M., Badano L.P., Belohlavek M., Cardim N.M., Derumeaux G., Galderisi M., Marwick T., Nagueh S.F., Sengupta P.P. (2011). Current and evolving echocardiographic techniques for the quantitative evaluation of cardiac mechanics: ASE/EAE consensus statement on methodology and indications endorsed by the Japanese Society of Echocardiography. J. Am. Soc. Echocardiogr..

[27] Yafasov M., Olsen F.J., Skaarup K.G., Lassen M.C.H., Johansen N.D., Lindgren F.L., Jensen G.B., Schnohr P., Mogelvang R., Sogaard P. (2024). Normal values for left atrial strain, volume, and function derived from 3D echocardiography: The Copenhagen City Heart Study. Eur. Heart J. Cardiovasc. Imaging.

[28] Galderisi M., Cosyns B., Edvardsen T., Cardim N., Delgado V., Di Salvo G., Donal E., Sade L.E., Ernande L., Garbi M. (2017). Standardization of adult transthoracic echocardiography reporting in agreement with recent chamber quantification, diastolic function, and heart valve disease recommendations: An expert consensus document of the European Association of Cardiovascular Imaging. Eur. Heart J. Cardiovasc. Imaging.

[29] Skaarup K.G., Lassen M.C.H., Johansen N.D., Olsen F.J., Lind J.N., Jorgensen P.G., Jensen G., Schnohr P., Prescott E., Sogaard P. (2022). Age- and sex-based normal values of layer-specific longitudinal and circumferential strain by speckle tracking echocardiography: The Copenhagen City Heart Study. Eur. Heart J. Cardiovasc. Imaging.

[30] Park J.H., Choi J.O., Park S.W., Cho G.Y., Oh J.K., Lee J.H., Seong I.W. (2018). Normal references of right ventricular strain values by two-dimensional strain echocardiography according to the age and gender. Int. J. Cardiovasc. Imaging.

[31] Baggish A.L., Battle R.W., Beaver T.A., Border W.L., Douglas P.S., Kramer C.M., Martinez M.W., Mercandetti J.H., Phelan D., Singh T.K. (2020). Recommendations on the Use of Multimodality Cardiovascular Imaging in Young Adult Competitive Athletes: A Report from the American Society of Echocardiography in Collaboration with the Society of Cardiovascular Computed Tomography and the Society for Cardiovascular Magnetic Resonance. J. Am. Soc. Echocardiogr..

[32] Pelliccia A., Caselli S., Sharma S., Basso C., Bax J.J., Corrado D., D’Andrea A., D’Ascenzi F., Di Paolo F.M., Edvardsen T. (2018). European Association of Preventive Cardiology (EAPC) and European Association of Cardiovascular Imaging (EACVI) joint position statement: Recommendations for the indication and interpretation of cardiovascular imaging in the evaluation of the athlete’s heart. Eur. Heart J..

[33] Caselli S., Montesanti D., Autore C., Di Paolo F.M., Pisicchio C., Squeo M.R., Musumeci B., Spataro A., Pandian N.G., Pelliccia A. (2015). Patterns of left ventricular longitudinal strain and strain rate in Olympic athletes. J. Am. Soc. Echocardiogr..

[34] Stobe S., Richter S., Seige M., Stehr S., Laufs U., Hagendorff A. (2020). Echocardiographic characteristics of patients with SARS-CoV-2 infection. Clin. Res. Cardiol..

[35] Croft L.B., Krishnamoorthy P., Ro R., Anastasius M., Zhao W., Buckley S., Goldman M., Argulian E., Sharma S.K., Kini A. (2021). Abnormal left ventricular global longitudinal strain by speckle tracking echocardiography in COVID-19 patients. Future Cardiol..

[36] Mahajan S., Kunal S., Shah B., Garg S., Palleda G.M., Bansal A., Batra V., Yusuf J., Mukhopadhyay S., Kumar S. (2021). Left ventricular global longitudinal strain in COVID-19 recovered patients. Echocardiography.

[37] Rajpal S., Tong M.S., Borchers J., Zareba K.M., Obarski T.P., Simonetti O.P., Daniels C.J. (2021). Cardiovascular Magnetic Resonance Findings in Competitive Athletes Recovering From COVID-19 Infection. JAMA Cardiol..

[38] Malek L.A., Marczak M., Milosz-Wieczorek B., Konopka M., Braksator W., Drygas W., Krzywanski J. (2021). Cardiac involvement in consecutive elite athletes recovered from COVID-19: A magnetic resonance study. J. Magn. Reson. Imaging.

[39] Brito D., Meester S., Yanamala N., Patel H.B., Balcik B.J., Casaclang-Verzosa G., Seetharam K., Riveros D., Beto R.J., Balla S. (2021). High Prevalence of Pericardial Involvement in College Student Athletes Recovering From COVID-19. JACC Cardiovasc. Imaging.

[40] Gherbesi E., Bergamaschi L., Cusmano I., Tien T.T., Paolisso P., Foa A., Pizzi C., Barosi A. (2022). The usefulness of speckle tracking echocardiography in identifying subclinical myocardial dysfunction in young adults recovered from mild COVID-19. Echocardiography.

[41] Hsiao J.F., Koshino Y., Bonnichsen C.R., Yu Y., Miller F.A., Pellikka P.A., Cooper L.T., Villarraga H.R. (2013). Speckle tracking echocardiography in acute myocarditis. Int. J. Cardiovasc. Imaging.

[42] Akkaya F., Yenercag F.N.T., Kaya A., Sener Y.Z., Bagci A. (2021). Long term effects of mild severity COVID-19 on right ventricular functions. Int. J. Cardiovasc. Imaging.

[43] Racz G., Takacs H., Kormanyos A., Polestyuk B., Borbas J., Gyenes N., Schvartz N., Nemeth G., Kincses Z.T., Sepp R. (2022). Screening for Myocardial Injury after Mild SARS-CoV-2 Infection with Advanced Transthoracic Echocardiography Modalities. Diagnostics.

[44] Dawkins T.G., Curry B.A., Wright S.P., Meah V.L., Yousef Z., Eves N.D., Shave R.E., Stembridge M. (2021). Right Ventricular Function and Region-Specific Adaptation in Athletes Engaged in High-Dynamic Sports: A Meta-Analysis. Circ. Cardiovasc. Imaging.

[45] Yaman B., Akpinar O., Kemal H.S., Cerit L., Sezenoz B., Acikgoz E., Duygu H. (2020). The beneficial effect of low-intensity exercise on cardiac performance assessed by two-dimensional speckle tracking echocardiography. Echocardiography.

[46] Goerlich E., Minhas A., Gilotra N., Barth A.S., Mukherjee M., Parziale A., Wu K.C., Hays A.G. (2021). Left Atrial Function in Patients with Coronavirus Disease 2019 and Its Association with Incident Atrial Fibrillation/Flutter. J. Am. Soc. Echocardiogr..

[47] ZeinElabdeen S.G., Sherif A., Kandil N.T., Altabib A.M.O., Abdelrashid M.A. (2023). Left atrial longitudinal strain analysis in long COVID-19 syndrome. Int. J. Cardiovasc. Imaging.

[48] Cuspidi C., Tadic M., Sala C., Gherbesi E., Grassi G., Mancia G. (2019). Left atrial function in elite athletes: A meta-analysis of two-dimensional speckle tracking echocardiographic studies. Clin. Cardiol..

[49] Blume G.G., McLeod C.J., Barnes M.E., Seward J.B., Pellikka P.A., Bastiansen P.M., Tsang T.S. (2011). Left atrial function: Physiology, assessment, and clinical implications. Eur. J. Echocardiogr..

